# Orchidaceae in Puglia (Italy): Consistency, Distribution, and Conservation

**DOI:** 10.3390/plants12112223

**Published:** 2023-06-05

**Authors:** Alessio Turco, Antonella Albano, Pietro Medagli, Saverio D’Emerico, Robert Philipp Wagensommer

**Affiliations:** 1 Department of Biological and Environmental Sciences and Technologies (DiSTeBA), Salento University, 73100 Lecce, Italy; alessio.turco@unisalento.it (A.T.); antonella.albano@unisalento.it (A.A.); pietro.medagli@unisalento.it (P.M.); 2 Aldo Moro University of Bari, 70125 Bari, Italy; sdeme@yahoo.it; 3 Faculty of Education, Free University of Bozen-Bolzano, 39042 Brixen-Bressanone, Italy

**Keywords:** flora, orchids, protected areas, taxonomical concerns

## Abstract

A study of the distribution of orchid species in Puglia, based on an analysis of 2084 bibliographic reports from 2000 to 2022, was carried out with the aim of revising and updating the information on the consistency of the Orchidaceae family in Puglia, with a special focus on assessing threatened species occurring inside and outside protected areas. The work presents a checklist of the Orchidaceae taxa (genera, species, and subspecies) found in the region, including observations on genera and species that present taxonomic challenges. A total of 113 taxa (i.e., species and subspecies), distributed across 16 genera, are listed in alphabetical order. The most representative genera were *Ophrys* (51 taxa), *Serapias* (15 taxa), and *Epipactis* (11 taxa). Additionally, 49 taxa (43.4%) were found to be endemic to Italy, with 21 of these, mostly belonging to the *Ophrys* genus, being exclusive to Puglia. Our study notes two different trends of distribution: a predominantly coastal distribution for orchid records located in southern Puglia (the Salento peninsula) and a more widespread distribution for the other provinces. Our study also shows that the greatest number of records locate orchids in protected areas with a positive correlation between their presence and habitats cited in Directive 92/43/EEC.

## 1. Introduction

The Orchidaceae family is a diverse group of plants and one of the most abundant among angiosperms, with over 28,000 accepted species and 763 genera. Many of these species are endemic to small areas and are considered rare and endangered [1,2]. Unfortunately, orchids are at a high risk of extinction, primarily due to the loss of their habitats and the effects of climate change [3]. However, due to their significant conservation value, Orchidaceae is a prominent plant family that receives attention from ecologists, biologists, and environmentalists worldwide [1]. In Europe, orchids are present in almost all habitats, and southern Europe, particularly the Mediterranean, has the highest species diversity. This region is considered the birthplace of certain orchid genera, such as *Ophrys* and *Serapias*, which boast remarkable diversity [3].

The Orchidaceae family is well represented in the native vascular flora of Italy, with 236 taxa (species and subspecies) within 27 genera. Among these, the *Ophrys* genus is particularly species-rich, with 103 taxa. When considering only Italian endemics, the Orchidaceae family is represented by 87 taxa, ranking below the Asteraceae, Plumbaginaceae, and Caryophyllaceae families. The *Ophrys* genus has 61 endemic taxa, with smaller numbers in the *Hieracium*, *Limonium*, and *Centaurea* genera [4]. The Puglia region has the highest orchid biodiversity, with 101 taxa out of 2552 native plants, followed by Tuscany (100 taxa) and Basilicata (97 taxa) [5]. In terms of Italian endemics, 31 species and subspecies of Orchidaceae occur in Puglia [4], many of which are related to eastern Mediterranean taxa, indicating its relative phytogeographical isolation with respect to other parts of southern peninsular Italy [6].

Puglia is an area of significant interest in terms of flora and vegetation due to its geological history and biogeographical location. The Orchidaceae family has been the focus of numerous botanical studies, including floristic surveys of specific areas and more in-depth investigations of the genetics and morphometry of certain taxa. These studies have often led to the discovery of new specific or subspecific taxa.

The earliest reports on the ancient local flora were conducted by researchers such as Baselice in 1812 for Gargano [7], Marinosci in 1870 [8], Groves in 1887 for Salento [9], and Palanza in 1900 for Bari area [10], all of which are considered pioneering. Later, more modern and systematic specialized studies of Mediterranean Orchidaceae were carried out, mainly by foreign authors. Nelson in 1968 [11]; the spouses Danesch (who described several new species and hybrids) in 1972 [12]; Baumann and Künkele in monographs on *Ophrys* and *Serapias* genera, respectively [13,14]; and Lorenz [15] for *Serapias* all made significant contributions. Additionally, cartographical studies were conducted by Gölz and Reinhard in 1982 for central-southern Puglia [16] and by Lorenz and Gembardt in 1987 for Gargano [17]. More recent research on orchids in Puglia has been published by Romolini and Souche [18], Delforge [19], and GIROS [20]. Souche in 2008 also provided a paper on the study of hybrid species [21].

The aim of this paper is to revise and update the information on the consistency of the Orchidaceae family in Puglia. This involves aligning the terminology with the latest taxonomy adopted by GIROS (Gruppo Italiano per la Ricerca sulle Orchidee Spontanee—Italian Group for Research on Spontaneous Orchids), examining the distribution in the region and recognizing the significance of conserving these species, which are frequently uncommon, endemic to restricted areas, and/or have their *locus classicus* in Puglia. These plants are employed as bioindicators in environmental management and the safeguarding of habitats.

## 2. Materials and Methods

### 2.1. Taxonomic References and Data Analysis

To evaluate the consistency and distribution of Orchidaceae in Puglia, an updated list of reports (Table S1) was created. Due to the high degree of nomenclature variation and the considerable number of new species, and in order to minimize attribution errors, records were sought only among new data published in the period 2000–2022, mainly, but not only, belonging to specialist papers (i.e., mainly from GIROS and GIROS Orch. Spont. Eur.). For each analysed paper, floristic list, occurring site, and geographic coordinates of the study site were noted. If the geographical coordinates were not given, they were subsequently obtained on the basis of other information on the occurring site cited. For all records, the geographic coordinates were converted into WGS 84 system coordinates. 

Table S1 contains all the previously mentioned information except for the coordinates of records that were used for cartographic processing, which were not included for conservation purposes and to avoid misuse.

For each taxon, the corotype was also added, taken either from GIROS [20] or from recent publications regarding new species. 

Overly generic records (e.g., *Serapias* without mentioning species attributes) were not considered in the dataset.

The checklist does not include the presence in Puglia of *Dactylorhiza maculata* (L.) Soó subsp. *fuchsii* (Druce) Hyl. [22,23,24] as, following Bartolucci et al. [4], it is considered an erroneous report. *Himantoglossum adriaticum* H.Baumann [25] is excluded for the same reason.

The reports of *Ophrys tardans* for Bosco Cuturi [26] and Bosco Difesa Grande [27] were considered to be *Ophrys expansa* (Lumare & Medagli) Biagioli, Kreutz, Lumare, Medagli & De Simoni, as stated by Lumare and Medagli [28].

All the reports for *Ophrys holosericea* subsp. *holosericea* (Burm. f.) Greuter were deleted from the checklist, in agreement with GIROS [20]. In addition, reports in which the identifications were recorded as “s.l.” (e.g., *O. fusca* s.l. and *O. lutea* s.l.) were deleted as they were considered too generic to be attributed to a species.

The reports of *Anacamptis papilionacea* subsp. *aegaea* (P.Delforge) L.Lewis & Kreutz for the Salento peninsula [29,30] were considered to be synonymous with *Anacamptis papilionacea* (L.) R.M.Bateman, Pridgeon & M.W.Chase due to the limited differences between these specimens and the type species.

The reports of *Ophrys passionis* Sennen for Puglia [31,32,33,34,35,36] were considered as *Ophrys garganica* E.Nelson ex O.Danesch et E.Danesch as *O. passionis* seems to be limited in its distribution to Spain and France [20].

The nomenclature of species follows Delforge [19] and the novelties in Biagioli et al. [37].

On the basis of geolocation data (and subsequent interpretation of satellite images) and other data reported in publications, the genera were correlated, applying our own expertise, with the habitats of Directive 92/43/EEC.

All maps were drawn using QGis ver. 3.22.16 LTR (www.qgis.org, accessed on 20 April 2023). Data analysis used all data from the database, including multiple reports from the same locality, assuming that the more times a species is reported, the greater its abundance in the investigated area.

### 2.2. Study Area

Puglia is an administrative region in southeastern Italy, covering an area of 19.541 km^2^. Its territory is mainly made up of plains (53.2%) and hills (45.3%), with mountains accounting for a very limited portion (1.5%) concentrated in the northern part of the region. Its coasts, washed by the Adriatic and Ionian seas, are partly sandy and partly rocky and have a total length of 840 km [38].

Puglia includes six provinces: Bari, BA (today, the metropolitan city of Bari); Foggia, FG; Barletta-Andria-Trani, BT; Taranto, TA; Brindisi, BR; and Lecce, LE. The largest province is Foggia (7008 km^2^), followed by Bari (3863 km^2^), Lecce (2799 km^2^), Taranto (2467 km^2^), Brindisi (1861 km^2^), and Barletta-Andria-Trani (1543 km^2^) [39].

## 3. Results

### 3.1. Floristic Data

Floristic data on the presence and distribution of orchids in Puglia were obtained by means of bibliographical research, which involved analysing 185 specialized papers containing 2084 reports (Table S1), with the aim of assessing the consistency and distribution of orchids in Puglia.

As listed in Table 1, orchids in Puglia are represented by 113 taxa (species and subspecies) within 16 genera. The genus with the most taxa is *Ophrys* (51), followed by *Serapias* (15) and *Epipactis* (11), while the genera with the least taxa are *Himantoglossum* and *Platanthera* (both 2), and *Coeloglossum*, *Epipogium*, *Gymnadenia*, *Limodorum*, *Neottia*, and *Spiranthes* (all one).

On the basis of the geolocation of each report (or, in cases in which the geographical coordinates were not given, the description of the province/municipality or area of discovery), the distribution of genera by province is given in Table 2.

As expected, in accordance with the results of Table 1, taking into account the total number of reports, *Ophrys* is the most frequently reported genus, accounting for 52.93% of records, followed by *Serapias* (19.04%) and *Anacamptis* (14.1%), while *Coeloglossum, Epipogium, Gymnadenia Spiranthes*, and *Neottia* are cited less frequently. Analytical data are presented in Table 3.

Considering the single reports obtained by bibliographical research, Table 4 lists the number of records for each genus by province. For the province of Bari, *Ophrys, Anacamptis*, and *Serapias* were the most frequently reported genera, as they were in other provinces excluding Barletta-Andria-Trani and Foggia. The latter, with the highest number of genera in the region, showed a similar trend but with the addition of the genera *Epipactis* and *Orchis*. In contrast, the province of Barletta-Andria-Trani had the lowest number of reports.

Considering the corotypes of the analysed orchids, there are 49 taxa (species and subspecies) endemic to Italy, with 620 records, and 3 subendemic taxa (i.e., with a restricted distribution that includes Italy and a small area beyond its border), with 23 records. With 36 taxa, *Ophrys* is the richest genus in terms of endemism, followed by *Serapias* (8 taxa), *Epipactis* (4 taxa), and *Anacamptis* (1 taxon). *Ophrys* also accounts for the only three subendemics.

Table 5 shows the number of records for each genus by province for taxa (species and subspecies) endemic to Italy. In this case, the province of Lecce has the highest number of endemic taxa, followed by the provinces of Foggia and Bari. As previously mentioned, all endemics belong to the genera *Ophrys* and *Serapias*, except in the province of Foggia, which also has endemics belonging to *Anacamptis* and *Epipactis*.

Table 6 shows the correlation between genera and habitats cited in Directive 92/43 EEC. Taxa within the genus *Ophrys* are seen to occupy the highest number of habitats (10), ranging from humid environments (i.e., habitat 1410) to xeric Mediterranean grasslands and forests. This is obviously also related to the high species diversity of this genus. In contrast, some genera are associated with a specific habitat group, as in the case of *Himantoglossum* and *Spiranthes*, which grow in xeric Mediterranean grasslands, and *Epipactis*, *Limodorum*, *Platanthera*, *Neottia*, *Cephalanthera*, *Coeloglossum*, and *Epipogium*, which prefer woody habitats.

### 3.2. Checklist of Orchids in Puglia

The updated checklist of Orchidaceae in Puglia includes 113 taxa (species and subspecies). Genera and species are alphabetically listed. “E” before the name of the species means endemic to Italy, while “SubE” means subendemic, i.e., with a restricted distribution that includes Italy and a small area beyond its border.
Genus***Anacamptis*** Rich.E***Anacamptis berica*** Doro [40]
***Anacamptis collina*** (Banks & Sol. ex Russell) R.M.Bateman, Pridgeon & M.W.Chase [22,27,31,32,41,42,43,44,45,46,47,48,49,50,51,52,53,54,55]
***Anacamptis fragrans*** (Pollini) R.M.Bateman [22,27,44,53,54,56,57,58,59,60,61,62,63,64,65,66,67,68,69,70,71,72,73,74]
***Anacamptis laxiflora*** (Lam.) R.M.Bateman, Pridgeon & M.W.Chase [27,59,64,65,75,76,77,78,79,80,81,82,83]
***Anacamptis morio*** (L.) R.M.Bateman, Pridgeon & M.W.Chase [22,27,29,30,31,32,33,34,42,43,44,45,46,47,48,49,50,52,53,54,56,58,60,61,62,63,64,65,67,68,70,73,74,75,78,80,83,84,85,86,87,88,89,90,91,92,93,94,95,96,97,98,99,100,101,102]
***Anacamptis palustris*** (Jacq.) R.M.Bateman, Pridgeon & M.W.Chase [56,64,65,75,77,79,80,103,104,105,106,107]
***Anacamptis papilionacea*** (L.) R.M.Bateman, Pridgeon & M.W.Chase [22,27,28,29,30,32,33,34,35,42,43,44,45,46,47,48,49,50,52,53,54,56,57,60,61,62,63,65,67,68,70,73,80,86,88,89,90,91,93,98,99,100,101,108,109,110,111,112,113]
***Anacamptis pyramidalis*** (L.) Rich. [22,27,28,35,42,44,47,49,52,53,56,58,60,61,62,63,64,66,68,70,71,72,73,78,80,84,86,88,89,90,92,93,94,95,98,101,103,108,110,113,114,115,116,117,118,119]Genus***Cephalanthera*** Rich.
***Cephalanthera damasonium*** (Mill.) Druce [23,24,27,84,117,120]
***Cephalanthera longifolia*** (L.) Fritsch [24,120]
***Cephalanthera rubra*** (L.) Rich. [24,121]Genus***Coeloglossum*** Hartm.
***Coeloglossum viride*** (L.) Hartm. [22]Genus***Dactylorhiza*** Neck. ex Nevski
***Dactylorhiza romana*** (Sebast.) Soó [22,27,46,61,111,122]
***Dactylorhiza saccifera*** (Brongn.) Soó [48,121,122]
***Dactylorhiza sambucina*** (L.) Soó [22]Genus***Epipactis*** Zinn
***Epipactis exilis*** P.Delforge [123]E***Epipactis garganica*** S.Hertel [124]
***Epipactis helleborine*** (L.) Crantz [24,27,117,121,125,126,127,128]E***Epipactis meridionalis*** H.Baumann & R.Lorenz [121,127]
***Epipactis microphylla*** (Ehrh.) Sw. [24,27,84,115,117,121,125,129]
***Epipactis muelleri*** Godfery [24,121]
***Epipactis neglecta*** (Kümpel) Kümpel [125]
***Epipactis palustris*** (L.) Crantz [103,130,131]
***Epipactis purpurata*** Sm. [123,127]E***Epipactis schubertiorum*** Bartolo, Pulv., & Robatsch [24,121]E***Epipactis umbrae*** (Kreutz, A.Rossini, Quitadamo, Turco & Medagli) Biagioli, Kreutz, A.Rossini, Quitadamo, Turco, Medagli & De Simoni [127]Genus***Epipogium*** Borkh.
***Epipogium aphyllum*** Sw. [121]Genus***Gymnadenia*** R.Br.
***Gymnadenia conopsea*** (L.) R.Br. [22,31,132]Genus***Himantoglossum*** Spreng.
***Himantoglossum hircinum*** (L.) Spreng. [22,27,42,46,90,102,111,117]
***Himantoglossum robertianum*** (Loisel.) P.Delforge [22,27,31,32,33,41,42,44,46,48,49,50,51,52,54,60,61,70,88,90,93,94,111,133,134]Genus***Limodorum*** Boehm.
***Limodorum abortivum*** (L.) Sw. [24,27,35,48,50,52,56,61,65,69,70,84,103,117,132]Genus***Neotinea*** Rchb.f.
***Neotinea lactea*** (Poir.) R.M.Bateman, Pridgeon & M.W.Chase [27,28,42,44,52,53,56,58,68,78,92]
***Neotinea maculata*** (Desf.) Stearn [93]
***Neotinea tridentata*** (Scop.) R.M.Bateman, Pridgeon & M.W.Chase [22,27,31,42,48,49,50,58,61,67,68,70,74,86,101,116,119]
***Neotinea ustulata*** (L.) R.M.Bateman, Pridgeon & M.W.Chase [22,27,42,49,67,74,91,101,113,114,116,119,135]Genus**Neottia** Guett.
***Neottia nidus-avis*** (L.) Rich. [22,23,24,48,117,121,136]Genus***Ophrys*** L.
***Ophrys apifera*** Huds. [22,27,35,46,48,61,65,69,70,75,76,79,80,81,82,83,101,103,104,108,111,115,116,119,120,132,137,138,139]E***Ophrys apulica*** (O.Danesch & E.Danesch) O.Danesch & E.Danesch [27,28,31,32,33,35,36,41,42,44,46,47,49,50,52,53,56,57,60,61,62,63,64,65,67,68,69,70,71,72,73,75,76,80,81,82,83,89,90,91,92,93,100,101,107,109,111,112,113,115,117,119,139,140,141,142,143,144,145,146,147,148,149,150]SubE***Ophrys archipelagi*** Gölz & H.R.Reinhard [41,68]
***Ophrys bertolonii*** Moretti [22,27,28,29,32,33,35,36,42,43,44,46,47,49,50,52,53,54,56,57,60,61,62,63,64,65,67,68,69,70,72,73,79,80,86,87,88,89,90,91,93,95,98,100,101,107,110,113,114,116,119,120,138,139,147,148,151,152,153]E***Ophrys bertoloniiformis*** O.Danesch & E.Danesch [22,31,54,68,70,99,140,154,155]E***Ophrys biscutella*** O.Danesch & E.Danesch [31,48,49,60,119,154,156,157]
***Ophrys bombyliflora*** Link [27,28,29,32,33,34,35,42,43,44,46,48,49,50,52,53,57,58,59,60,61,62,63,64,65,66,67,68,69,70,71,73,75,76,78,79,80,81,82,83,87,88,89,90,91,98,100,101,109,110,111,112,114,119,138,139,142,143,147,150,152,154,158,159,160]E***Ophrys brutia*** P.Delforge [36,50,68,91,142,148,158,161,162]SubE***Ophrys calocaerina*** Devillers-Tersch. & Devillers [163]
***Ophrys candica*** (E.Nelson ex Soó) H.Baumann & Künkele [44,47,52,53,56,62,63,65,72,92,98,107,115,143,147,159,164,165,166]E***Ophrys celiensis*** O.Danesch & E.Danesch [27,35,50,61,70,93,100,111,113,139,155,167]E***Ophrys cinnabarina*** Romolini & Soca [27,31,35,46,69,101,111,116,139,140,144]E***Ophrys classica*** Devillers-Tersch. & Devillers [31,42,48,53,56,68,70,74,86,90,103,107,112,114,117,140,142,144,145,148,154,158,160,162]E***Ophrys conradiae*** Melki & Deschatres [27,35,46,48,67,89,101,111,139,168,169,170]SubE***Ophrys corsica*** Soleirol ex G.Foelsche & W.Foelsche [27,33,36,48,49,53,70,91,98,101,110,147,154,171]
***Ophrys eleonorae*** Devillers-Tersch. & Devillers [31,50,54,131,161,172]E***Ophrys exaltata*** Ten. [27]E***Ophrys expansa*** (Lumare & Medagli) Biagioli, Kreutz, Lumare, Medagli & De Simoni [26,28,52,53,112,152]
***Ophrys funerea*** Viv. [22,27,60,137,172,173]E***Ophrys garganica*** E.Nelson ex O.Danesch & E.Danesch [22,27,28,29,31,32,33,34,35,36,41,42,44,46,48,50,53,54,56,57,59,64,65,66,67,68,70,71,75,76,78,80,81,87,88,89,90,91,93,94,99,100,101,109,111,112,113,114,117,119,131,138,139,142,143,144,145,148,150,152,155,158,160,161,163]E***Ophrys gracilis*** (Büel, O.Danesch & E.Danesch) Englmaier [27,31,35,48,89,101,119,132,140,157]E***Ophrys gravinensis*** D’Alonzo [5,49,67,91,114]
***Ophrys incubacea*** Bianca [27,28,32,33,34,35,42,44,46,48,49,50,52,53,57,58,60,61,62,63,64,66,67,68,69,70,71,75,76,78,80,81,87,88,89,90,91,93,95,97,100,101,107,109,110,111,112,113,114,117,120,133,138,139,142,143,145,148,149,150,152,158]E***Ophrys ingrassiae*** (Dura, Turco, Gennaio & Medagli) Biagioli, Kreutz, Dura, Turco, Gennaio, Medagli, & De Simoni [5,61,72,93]E***Oprhys japigiae*** Turco, D’Emerico, Dura, Gennaio & Medagli [172]
***Ophrys lacaitae*** Lojac. [48,69,89,101,116,119,132,138,140,155,157,174]E***Ophrys lojaconoi*** P.Delforge [27,31,48,50,54,140,144,161,172,173,175]E***Ophrys lucana*** P.Delforge, Devillers-Tersch. & Devillers [27,61,70,116,120,133,172,176]E***Ophrys lucifera*** Devillers-Tersch. & Devillers [27,41,109,118,158,173]E***Ophrys lupercalis*** Devillers-Tersch. & Devillers [65,109,118,141,161,172,175]
***Ophrys lutea*** Cav. [27,28,29,32,33,34,35,42,43,44,48,50,53,57,58,60,61,63,64,65,68,69,70,71,73,80,84,85,86,88,91,94,95,97,98,110,112,119,133,138,139,142,147,152,154,158]E***Ophrys mateolana*** Medagli, D’Emerico, Bianco & Ruggiero [27,42,46,49,68,88]E***Ophrys mattinatae*** Medagli, A.Rossini, Quitadamo, D’Emerico & Turco [5,177]E***Ophrys minipassionis*** Romolini & Soca [27,31,48,70,140,154]E***Ophrys montis-gargani*** (Van de Vijver & W.Looken) Biagioli, Kreutz & De Simoni [17,144,178]E***Ophrys murgiana*** Cillo, Medagli & Margh. [5,33,87]
***Ophrys neglecta*** Parl. [22,27,28,29,31,32,34,35,42,43,44,46,48,49,50,52,54,56,57,58,60,61,62,63,64,65,67,68,69,70,71,72,73,80,88,89,91,92,93,94,95,98,99,100,101,102,103,109,110,111,112,113,114,118,119,120,138,139,140,143,147,148,152,154,158,163,165,179]E***Ophrys panormitana*** (Tod.) Soó [180]E***Ophrys paolina*** (V.Liverani & Romolini) Biagioli, Kreutz, V.Liverani, Romolini & De Simoni [48,69,89,101]E***Ophrys parvimaculata*** (O.Danesch & E.Danesch) Paulus & Gack [27,36,42,46,48,49,52,67,68,69,70,88,89,91,93,98,109,113,115,141,142,147,148,149,157,158,159,161,163,181]E***Ophrys peucetiae*** Lozito, D’Emerico, Medagli & Turco [5,109,141]E***Ophrys pinguis*** Romolini & Soca [182]E***Ophrys pollinensis*** E.Nelson ex Devillers-Tersch. & Devillers [93]E***Ophrys promontorii*** O.Danesch & E.Danesch [22,27,89,155]E***Ophrys pseudomelena*** Turco, Medagli & D’Emerico [5,53,109,141]
***Ophrys sicula*** Tineo [22,27,32,33,36,41,42,44,47,48,49,50,53,54,56,57,59,60,61,63,64,67,68,70,71,73,78,89,90,91,93,94,98,99,100,109,110,111,112,114,118,119,138,147,152,154]E***Ophrys sipontensis*** (Gumpr.) O.Danesch & E.Danesch [27,31,33,36,48,49,50,54,91,144,149]
***Ophrys speculum*** Link [22,33,47,113,145,155,183]E***Ophrys tardans*** O.Danesch & E.Danesch [5,53,57,95,110,138,146,151,153,164,166,179,184,185]E***Ophrys tarentina*** Gölz & H.R.Reinhard [27,47,52,53,68,70,84,94,108,112,148,150,163]E***Ophrys tarquinia*** P.Delforge [186]Genus***Orchis*** L.
***Orchis anthropophora*** (L.) All. [22,27,32,33,42,43,48,49,60,61,69,88,89,91,117,120]
***Orchis italica*** Poir. [22,27,32,42,48,50,52,58,60,61,66,69,70,88,89,90,94,99,101,113,116,117,119,120]
***Orchis mascula*** (L.) L. [120]
***Orchis pauciflora*** Ten. [22,23,74]
***Orchis provincialis*** Balb. ex Lam. & DC. [23,69]
***Orchis purpurea*** Huds. [23,27,113,117,120]
***Orchis quadripunctata*** Cirillo ex Ten. [22,48,101,116]
***Orchis simia*** Lam. [27,46,111,116,187]Genus***Platanthera*** Rich.
***Platanthera bifolia*** (L.) Rich. [188]
***Platanthera chlorantha*** (Custer) Rchb. [22,27,35,46,48,50,61,69,70,84,111,121,132,189]Genus***Serapias*** L.E***Serapias apulica*** (H.Baumann & Künkele) P.Delforge [5,27,54,57,58,64,78,81,92,95,190,191,192,193,194,195,196,197,198,199,200,201,202]E***Serapias ausoniae*** Gennaio & Pellegrino [203]
***Serapias bergonii*** E.G.Camus [27,34,52,53,57,60,61,63,64,68,70,73,81,82,92,95,97,142,158,159,190,192,193,194,196,197,198,199,200,201]E***Serapias brundisina*** (Lumare & Medagli) Biagioli, Kreutz, Lumare, Medagli & De Simoni [95,97,202]
***Serapias cordigera*** L. [27,34,35,46,48,50,53,64,69,70,72,73,78,79,92,101,111,116,119,142,190,192,193,194,196,198,199,200,201,204]E***Serapias guadinae*** Lumare, Medagli & Biagioli [201]
***Serapias lingua*** L. [22,27,31,32,35,46,48,49,50,53,56,60,61,62,63,64,65,68,69,70,72,73,78,80,83,88,89,91,92,95,96,97,100,111,114,158,192,193,194,196,197,199,200]E***Serapias messapica*** (Lumare & Medagli) Biagioli, Kreutz, Lumare, Medagli & De Simoni [95,202]E***Serapias neretina*** (Lumare & Medagli) Biagioli, Kreutz, Lumare, Medagli & De Simoni [52,95,202]
***Serapias parviflora*** Parl. [22,27,32,33,35,46,47,49,52,53,56,57,60,61,63,64,65,68,69,70,72,73,75,79,80,81,82,83,90,91,92,95,96,97,98,109,111,113,133,159,192,193,194,196,198,199,200,203,205]
***Serapias politisii*** Renz [52,53,57,62,63,64,72,73,75,76,92,95,96,98,142,158,159,190,192,193,194,196,199,200,201,204,205]E***Serapias sallentina*** (Lumare & Medagli) Biagioli, Kreutz, Lumare, Medagli & De Simoni [92,194,198,204]E***Serapias uxentina*** Gennaio [81,82,83]
***Serapias vomeracea*** (Burm.f.) Briq. subsp. *vomeracea* [22,27,32,33,35,42,43,46,48,49,50,53,61,63,64,65,67,69,70,71,73,74,76,79,81,83,85,89,90,91,92,95,96,100,101,102,103,111,113,114,115,116,118,119,120,142,158,190,192,193,194,196,198,199,200,201,204,206,207]
***Serapias vomeracea*** (Burm.f.) Briq. subsp. ***longipetala*** (Ten.) Baumann & Künkele [53,57,62,63,64,81,82,92,95,96,97,142,192,193,194,196,198,199,200,201,204,208]Genus***Spiranthes*** Rich.
***Spiranthes spiralis*** (L.) Chevall. [27,42,44,53,61,70]

## 4. Discussion

Our study presents the findings of bibliographical research on orchids in Puglia covering the years from 2000 to 2022. We opted to focus on data from the last two decades, mainly from specialized journals, because they provided more precise determinations, particularly for taxonomically critical genera such as *Ophrys*, *Serapias*, and *Epipactis*. Additionally, there have been ongoing nomenclatural changes, and other taxa have been added in recent years. In this study, we considered not only the presence of orchids but also their location, distribution, and preferred habitats.

Thanks to this research project, the orchidological biodiversity of Puglia has been updated to include 113 taxa (species and subspecies), of which 49 (43.4%) are endemic to Italy. These numbers exceed those reported in previous studies. For instance, the authors of [4] reported only 31 endemic taxa, and those of [209] reported 42 taxa, while our research found 52 endemic/subendemic taxa. The same trend was observed in the overall number of species. Among the endemics, 21 taxa (species and subspecies), mostly belonging to the genus *Ophrys*, are exclusive to Puglia. This growth is also consistent with what was recently asserted in [210].

**Figure 1:**
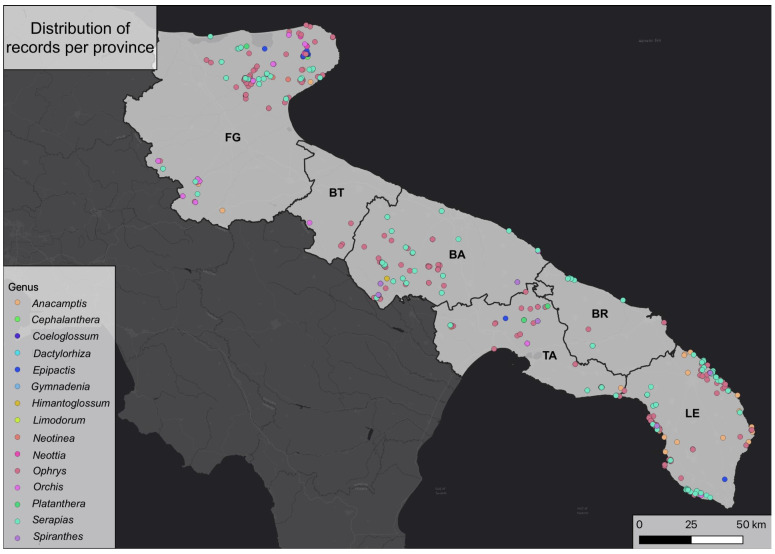
Orchid genera across Puglia based on the examined 2084 records.

The distribution of records was used to draw up a map of orchids across Puglia using geolocation data extracted from publications and other information that helped us to geolocate the study areas (Figure 1, Table S1). As can be seen, most of the records for the Salento peninsula are situated near the coastline, indicating that a significant portion of the biodiversity of this area in terms of environments and habitats is concentrated there. Indeed, the Salento peninsula is characterized mainly by urban areas and agroecosystems, which have eroded almost all the biodiversity located inland. In contrast, the records are more dispersed in the rest of Puglia, where woody environments are more extensive and abundant in addition to coastal and transitional environments.

**Figure 2:**
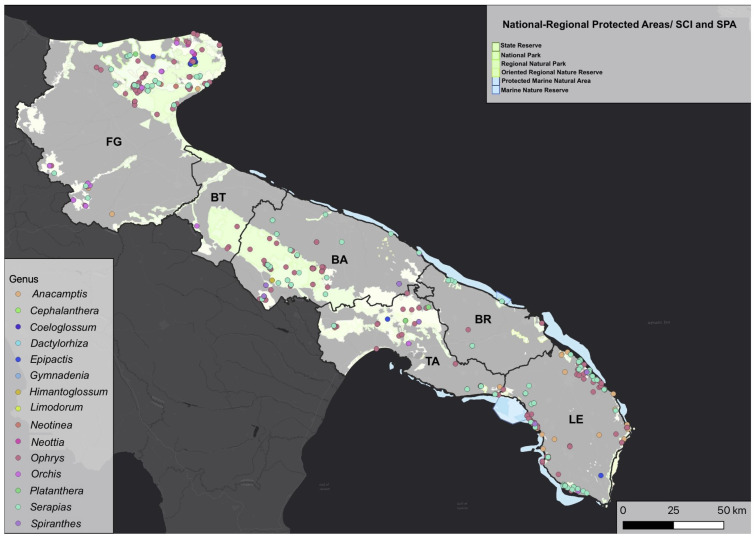
Distribution of orchid genera (based on the examined 2084 records) and protected areas across Puglia.

It is worth noting that a significant majority of orchid records are found within protected areas such as parks, SCIs, and SACs (Figure 2). This reinforces the significance of these areas not only for conservation purposes but also as areas that can be used for research activities by naturalists and scientists.

Based on our data, the province with the highest number of records is Lecce, followed by Bari, Foggia, Taranto, Brindisi, and Barletta-Andria-Trani (Table 4). The limited availability of data for the BT province can not only be attributed to its recent establishment in 2009 but also to its small size and because it has clearly been poorly investigated by botanists and naturalists.

**Figure 3:**
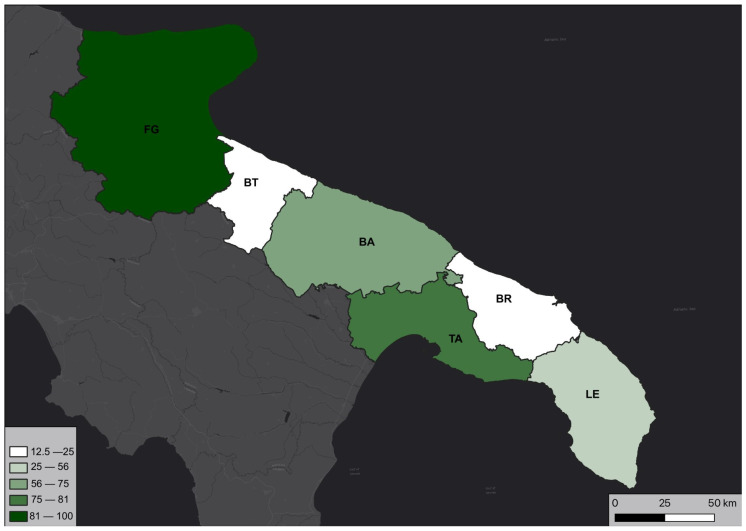
Percentage (%) of orchid genera in Puglia present in each province.

Referring to Table 2 and considering Lorenz and Gembardt’s [17] published report on *Spiranthes spiralis* in the province of Foggia, the distribution of genera by province is presented in Figure 3.

**Figure 4:**
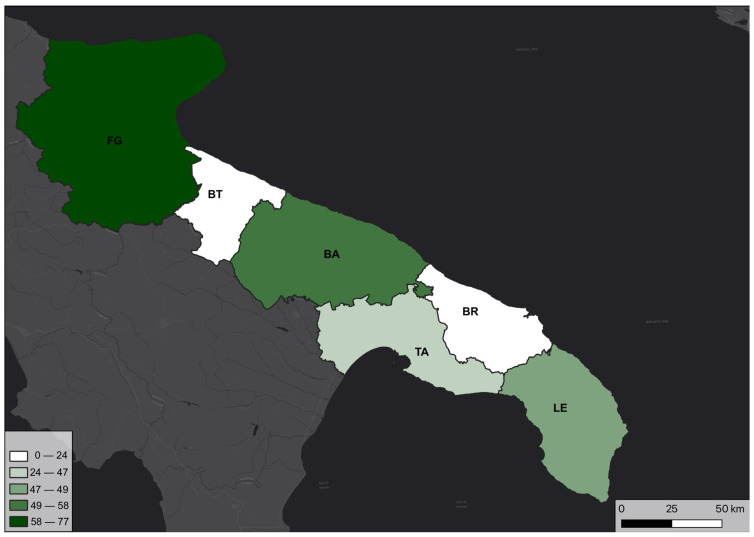
Percentage (%) of orchid taxa (species and subspecies) in Puglia by province.

The trend observed in the distribution of species and reports by province seems to differ from the distribution of genera depicted in Figure 3. Despite having a lower number of genera, the provinces of Bari and Lecce exhibit a higher richness of taxa (species and subspecies) than the province of Taranto. Meanwhile, Foggia maintains its status as the province with the highest species richness (Figure 4).

In terms of species reports, the province of Bari has the highest number of reports for the genera *Ophrys* and *Himantoglossum*, while the province of Lecce has the majority of reports for the genera *Serapias* and *Anacamptis,* and the province of Foggia has the highest number of reports for the genera *Orchis* and *Epipactis*.

Puglia serves as the southernmost boundary for certain Italian species such as *Anacamptis palustris*, *Ophrys funerea*, *O. lucifera*, *O. parvimaculata*, and *O. minipassionis*. However, it also marks the northernmost boundary for other taxa such as *Epipactis aspromontana*, *E. schubertiorum*, *Ophrys celiensis*, *O. sipontensis*, and *O. tarentina*, as stated by Pezzetta [209].

## 5. Conclusions

In conclusion, our study provides an updated and comprehensive checklist of orchid species and subspecies found in Puglia, including taxonomic observations and assessments of threatened species within and outside of protected areas. Our study, based on a thorough analysis of bibliographic reports spanning over two decades, contributes significantly to the understanding of Orchidaceae diversity in the region.

The checklist of 113 taxa (species and subspecies) across 16 genera also provides a comprehensive overview, highlighting taxonomic challenges and the prevalence of certain genera such as *Ophrys*, *Serapias*, and *Epipactis* compared to other genera. The presence of 49 taxa endemic to Italy, with a substantial number exclusive to Puglia, underscores the region’s significance for orchid biodiversity and conservation. In this context, it must be highlighted that all new species novelties were added in the checklist, including that of *Ophrys panormitana* [180], for which further investigations should be made to exclude the hybridogenic origin of the plants, as only one plant was found with several tufts of vegetative origin, which would suggest a morphological convergence rather than the presence of a new species, in this case, endemic to Sicily.

The distribution patterns reveal distinct trends, with a strong coastal preference in the south of Puglia and a more widespread distribution in other provinces. Importantly, this study underscores the importance of protected areas as critical habitats for orchid populations. Overall, this research enhances our knowledge of Puglia’s orchid flora and emphasizes the need for conservation efforts to safeguard these valuable plant species.

## Figures and Tables

**Table 1:** Genera and number of taxa (species and subspecies) per genus of orchids in Puglia derived from analysis of 2084 records.

Genus	Number of Taxa	Genus	Number of Taxa
*Anacamptis*	8	*Limodorum*	1
*Cephalanthera*	3	*Neotinea*	4
*Coeloglossum*	1	*Neottia*	1
*Dactylorhiza*	3	*Ophrys*	51
*Epipactis*	11	*Orchis*	8
*Epipogium*	1	*Platanthera*	2
*Gymnadenia*	1	*Serapias*	15
*Himantoglossum*	2	*Spiranthes*	1

**Table 2:** Orchid genera by province, with the percentage of the total orchid genera, derived from analysis of 2084 records.

Province	Number of Genera	%
BA	12	75
BR	4	25
BT	2	12.5
FG	15	94
LE	9	56
TA	13	81

**Table 3:** Number of reports of Orchidaceae genera, with the percentage of the total orchid genera, based on the examined 2084 records.

Genus	Number of Reports	%
*Anacamptis*	294	14.1
*Cephalanthera*	15	0.72
*Coeloglossum*	1	0.05
*Dactylorhiza*	10	0.5
*Epipactis*	46	2.2
*Epipogium*	1	0.05
*Gymnadenia*	3	0.14
*Himantoglossum*	39	1.87
*Limodorum*	15	0.72
*Neotinea*	52	2.49
*Neottia*	8	0.38
*Ophrys*	1103	52.93
*Orchis*	77	3.69
*Platanthera*	16	0.77
*Serapias*	397	19.04
*Spiranthes*	7	0.33
**Total**	**2084**	**100**

**Table 4:** Reports for each genus by province, with the percentage of the provincial total, based on the examined 2084 records.

Genus	BA		BR		BT		FG		LE		TA	
	N	%	N	%	N	%	N	%	N	%	N	%
*Anacamptis*	74	13.2	20	29.0			50	9.2	110	15.5	40	20.3
*Cephalanthera*	1	0.2					13	2.4			1	0.5
*Coeloglossum*							1	0.2				
*Dactylorhiza*	3	0.5					5	0.9			2	1.0
*Epipactis*	2	0.4					39	7.2	3	0.4	2	1.0
*Epipogium*							1	0.2				
*Gymnadenia*							3	0.6				
*Himantoglossum*	21	3.7					11	2.0	2	0.3	5	2.5
*Limodorum*	4	0.7					6	1.1	2	0.3	3	1.5
*Neotinea*	22	3.9	1	1.4			16	2.9	7	1.0	6	3.0
*Neottia*							7	1.3			1	0.5
*Ophrys*	332	59.1	28	40.6	1	50.0	300	55.2	331	46.6	111	56.3
*Orchis*	27	4.8			1	50.0	44	8.1	1	0.1	4	2.0
*Platanthera*	7	1.2					6	1.1			3	1.5
*Serapias*	65	11.6	20	29.0			41	7.6	253	35.6	18	9.1
*Spiranthes*	4	0.7							2	0.3	1	0.5
**Total**	**562**	**100**	**69**	**100**	**2**	**100**	**543**	**100**	**711**	**100**	**197**	**100**

**Table 5:** Distribution of records of taxa (species and subspecies) endemic to Italy, by genus and province, based on 620 reports.

Genus	BA		BR		BT		FG		LE		TA	
	N	%	N	%	N	%	N	%	N	%	N	%
*Anacamptis*							1					
*Epipactis*							18					
*Ophrys*	171		7		1		159		148		56	
*Serapias*	4		5				5		44		1	
**Total**	**175**	**100**	**12**	**100**	**1**	**100**	**183**	**100**	**192**	**100**	**57**	**100**

**Table 6:** Presence of orchids in habitats cited in Directive 92/43/EEC (1410 Mediterranean salt meadows; 2270* wooded dunes with pines; 5420 *Sarcopoterium spinosum* phryganas; 6210*, 6220*, and 62A0 xeric Mediterranean grasslands; 91H0* and 91M0 Pannonian woods; 9210* Apennine beech forests; 9250 *Quercus trojana* woods; 9260 *Castanea sativa* woods; 9340 *Quercus ilex* and *Quercus rotundifolia* forests; 9540 Mediterranean pine forests with endemic Mesogean pines).

Natura 2000 Code	1410	2270*	5420	6210*	6220*	62A0	91H0*	91M0	9210*	9250	9260	9340	9540
*Anacamptis*	•		•	•	•	•				•			
*Cephalanthera*									•				
*Coeloglossum*								•					
*Dactylorhiza*				•		•			•				
*Epipactis*									•		•	•	
*Epipogium*									•				
*Gymnadenia*													•
*Himantoglossum*				•	•	•							
*Limodorum*									•	•		•	
*Neotinea*				•	•	•			•				•
*Neottia*									•			•	
*Ophrys*	•	•		•	•	•	•		•	•		•	•
*Orchis*				•		•					•		•
*Platanthera*										•	•	•	
*Serapias*	•			•	•	•							•
*Spiranthes*					•	•							

## Data Availability

Data are contained in the article, including the supplementary material.

## Supplementary Materials

The following supporting information can be downloaded at https://www.mdpi.com/article/10.3390/plants12112223/s1. Table S1: Examined reports of Orchidaceae in Puglia (Italy).
